# Supplementary material for: A R-loop sensing pathway mediates the relocation of transcribed genes to nuclear pore complexes
Source: Nat Commun. 2023 Sep 20;14:5606. doi: 10.1038/s41467-023-41345-z (PMC10511428; doi:10.1038/s41467-023-41345-z)
Supplement: Supplementary file 1 — Supplementary Information [file 41467_2023_41345_MOESM1_ESM.pdf]

Supplementary Information

A R-loop sensing pathway mediates the relocation of transcribed genes to nuclear pore complexes

Arianna Penzo, Marion Dubarry, Clémentine Brocas, Myriam Zheng, Raphaël M. Mangione, Mathieu Rougemaille, Coralie Goncalves, Ophélie Lautier, Domenico Libri, Marie-Noëlle Simon, Vincent Géli, Karine Dubrana & Benoit Palancade

Includes:

Supplementary Figures 1-5

Supplementary Tables 1-4

Supplementary References

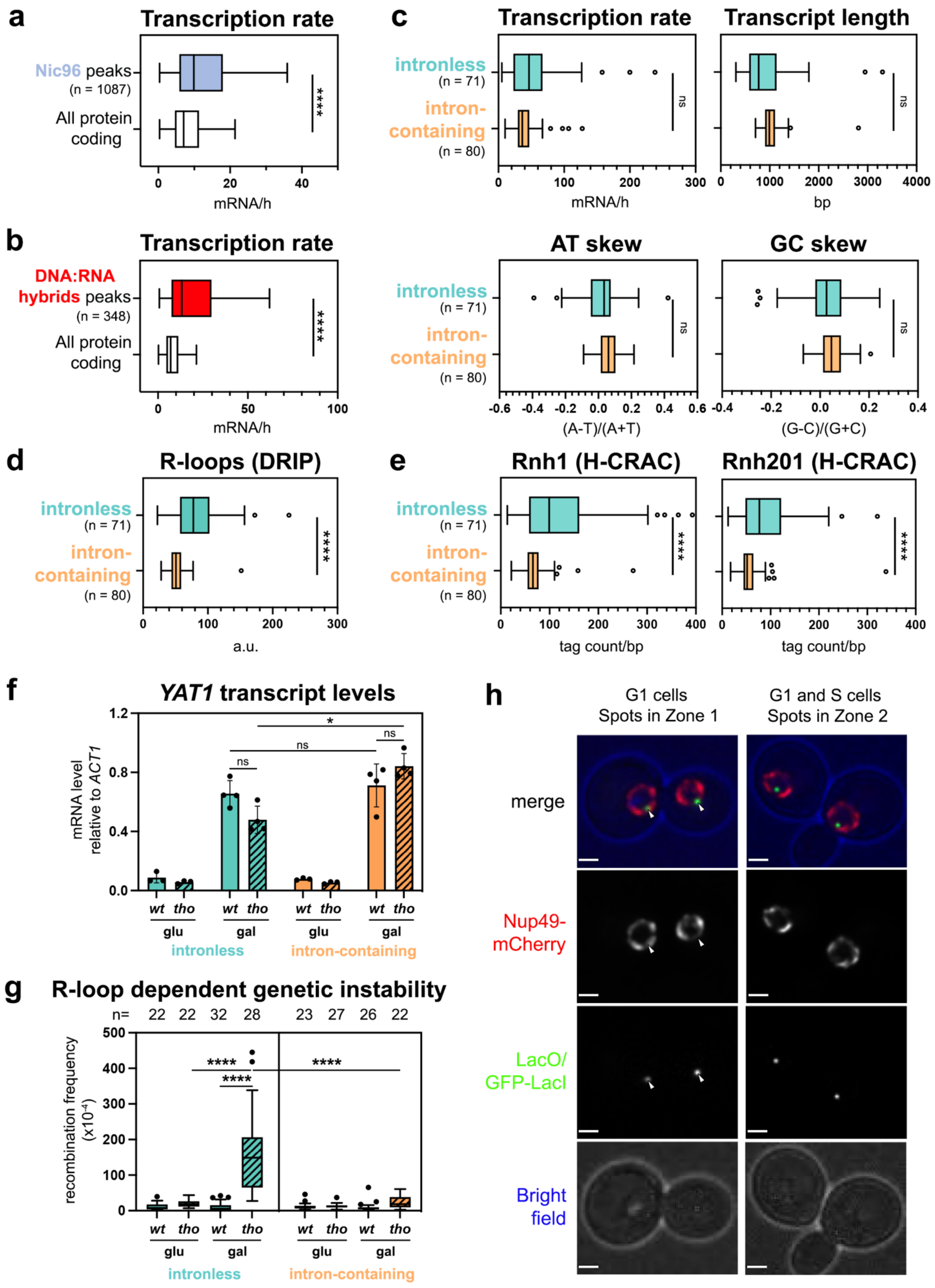

Supplementary Figure 1. Validation of the gene datasets and reporter systems used to analyze the relationships between NPC association and R-loop levels. See legend on next page.

Supplementary Figure 1. Validation of the gene datasets and reporter systems used to analyze the relationships between NPC association and R-loop levels. a-b, Transcription rates (mRNA/hr)[1] for the genes associated with Nic96 peaks (this study), DNA:RNA hybrid peaks [2] or all protein-coding genes. The number of considered peaks is indicated. Outliers identified according to Tukey's definition are not represented on this scale but have been included in statistical analyses. Statistical test: two-sided Mann-Whitney-Wilcoxon test; ****, $p<10^{-4}$. **c,** Transcription rates (mRNA/hr)[1], transcript length (pre-mRNA, bp), AT-skew and GC-skew for the intronless and intron-containing highly-expressed genes as previously defined[3] (listed in Supplementary Table 1). Statistical test: two-sided Mann-Whitney-Wilcoxon test**. d-e,** R-loops levels for the same groups of intronless and intron-containing highly-expressed genes as scored by DNA:RNA hybrid immunoprecipitation (DRIP[2]; **d**), RNase H1 CRAC[4] (**e**, left panel) and RNase H2 CRAC[4] (**e**, right panel). Statistical test: two-sided Mann-Whitney-Wilcoxon test; ****, $p<10^{-4}$. **f,** qPCR-based quantification of *YAT1* mRNA levels from *wt* or *tho* (*mft1*Δ) mutant cells grown in glycerol-lactate medium and further treated with either glucose (glu) or galactose (gal) for 5h (values normalized to *ACT1* RNA levels and relative to *wt*; mean±SD, n=3 independent experiments in glucose, 4 in galactose). Statistical test: two-sided Mann-Whitney-Wilcoxon test; *, $p=2.86 \times 10^{-2}$. **g,** Recombination frequencies (fraction of Leu+ prototrophs, $\times 10^{-4}$; n refers to the number of biologically independent cultures assessed for each strain/condition) were calculated as described in Methods for *wt* or *tho* (*mft1*Δ) mutant cells carrying *YAT1* transgenes, grown in glycerol-lactate medium and further treated with either glucose (glu) or galactose (gal) for 5h. Boxes extend from the 25th to 75th percentiles, with the median displayed as a line. The whiskers mark the minimum and maximum, displaying as individual points the values that fall outside of 1.5 time the inter-quartile range of the first or third quartile (Tukey's definition). Statistical test: two-sided Mann-Whitney-Wilcoxon test; ****, $p<10^{-4}$. **h,** Single channel images illustrating the principle of the zoning assay (*YAT1* tagged locus, Fig. 1g-h). Arrowheads point to loci localized in zone 1, and thus scored as peripheral. Note their colocalization with the Nup49-mCherry signal, even if not within the most prominent NPC patches. Scale bar = 1µm. Source data are provided as a Source Data file.

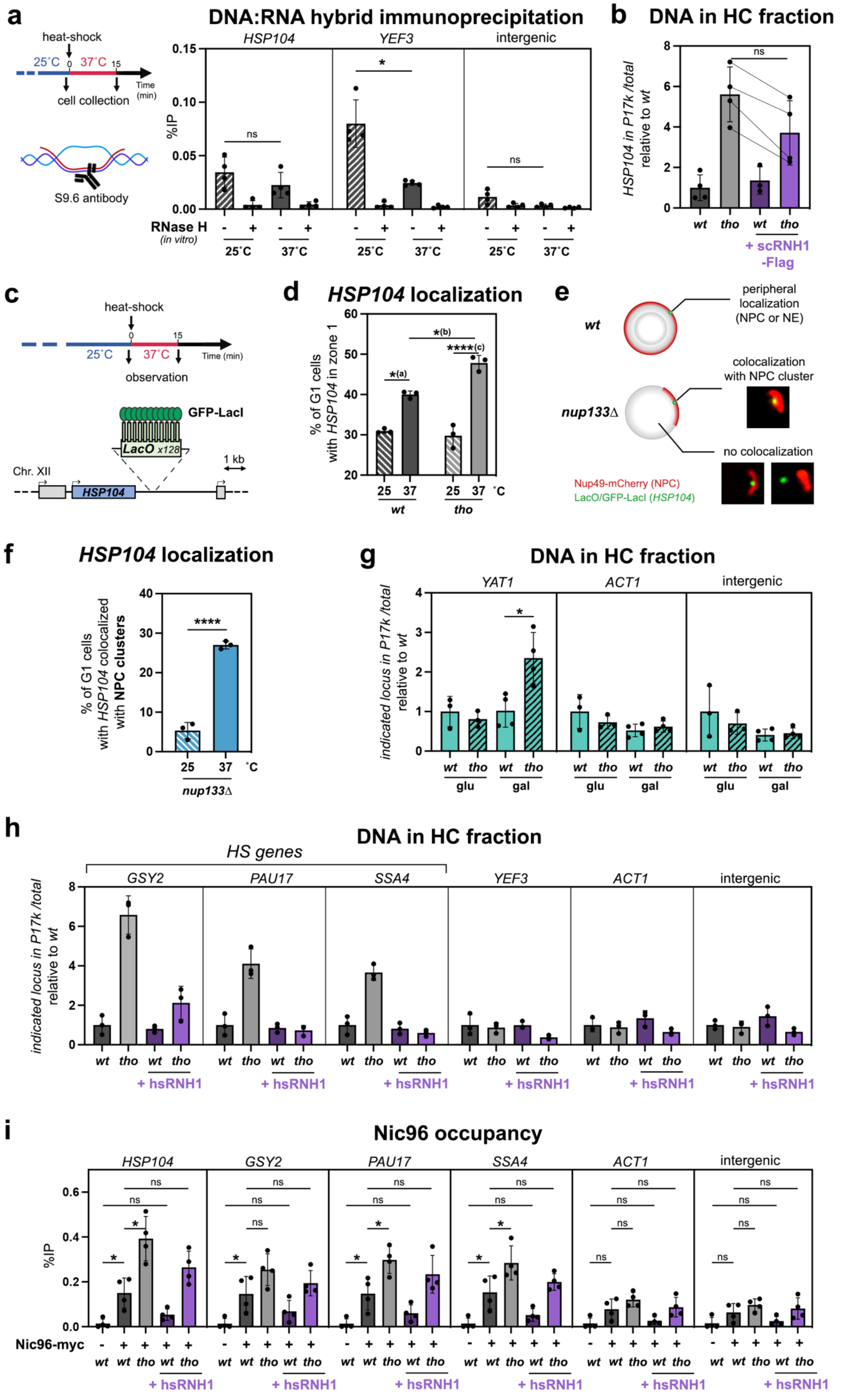

Supplementary Figure 2. Characterization of R-loop formation and relocation for heat shock loci. See legend on next page.

Supplementary Figure 2. Characterization of R-loop formation and relocation for heat shock loci. a, left panel, time-line of the heat shock and cell collection procedure used for DNA:RNA hybrid immunoprecipitation (DRIP) experiments. Right panel, DNA:RNA hybrid detection by DRIP-qPCR in *wt* cells grown at 25°C or heat-shocked at 37°C for 15min (% of IP; n=4 independent experiments). When indicated, DNA extracts were treated with RNase H *in vitro* prior to immunoprecipitation. Statistical test: two-sided Mann-Whitney-Wilcoxon test; *, $p=2.86 \times 10^{-2}$. **b**, qPCR-based quantification of the amount of DNA from the indicated loci in heavy chromatin (HC) fractions from *wt* or *tho* (*mft1*Δ) mutant cells transformed with either an empty vector or the *tetOFF-scRNH1-Flag* construct (+scRNH1), and heat-shocked at 37°C for 15min (% of the indicated locus in P17K relative to total [S17K+P17K]; mean±SD, n=4 independent experiments or 3 for wt+scRNH1, relative to *wt*). Statistical test: two-sided Mann-Whitney-Wilcoxon test. **c**, Schematic representation of the time-line of the procedure and of the tagged genomic *HSP104* locus used for microscopy experiments. **d,** Fraction of G1 cells (%) showing *HSP104* in zone 1 (mean±SD, n=3 independent experiments), in the indicated strains grown at 25°C or heat shocked at 37°C for 15min. Statistical test: two-sided Fisher's exact test; P-values were calculated on the total number of counted cells (between 326 and 409 cells/condition); (a), $p=1.59 \times 10^{-2}$; (b), $p=2.45 \times 10^{-2}$; (c), $p=4.96 \times 10^{-7}$. Values are the same as in Fig. 3h. **e**, Representative images of the position of the tagged genomic *HSP104* locus (in green) in *nup133*Δ cells with respect to NPC clusters (visualized owing to the expression of the Nup49-mCherry nucleoporin). **f,** Fraction of G1 cells (%) showing colocalization of *HSP104* with the NPC clusters in *nup133*Δ cells grown at 25°C or heat-shocked at 37°C for 15min (mean±SD, n=3 independent experiments). Statistical test: two-sided Fisher's exact test; the P-value was calculated on the total number of counted cells (25°C: 100; 37°C: 112); ****, $p=3.27 \times 10^{-5}$. **g**, qPCR-based quantification of the amount of DNA from the indicated loci in heavy chromatin (HC) fractions from *GAL-YAT1* cells, either *wt* or *tho* (*mft1*Δ), grown in glycerol-lactate medium and further treated with either glucose (glu) or galactose (gal) for 5h (% of the indicated locus in P17K relative to total [S17K+P17K]; mean±SD, n=3 independent experiments in glucose or 4 in galactose, relative to *wt*). Statistical test: two-sided Mann-Whitney-Wilcoxon test; *, $p=2.86 \times 10^{-2}$. Note that the co-fractionation phenotype is less pronounced for *YAT1* than for the HS genes, possibly in line with the particular high-frequency promoter firing in the latter case[5]. **h**, qPCR-based quantification of the amount of DNA from the indicated loci in heavy chromatin (HC) fractions from *wt* or *tho* (*mft1*Δ) mutant cells transformed with either an empty vector or the *GPD-hsRNH1* construct (+hsRNH1), and heat-shocked at 37°C for 15min (% of the indicated locus in P17K relative to total [S17K+P17K]; mean±SD, n=3 independent experiments, relative to *wt*). **i,** Nic96-myc enrichment was analyzed at the indicated loci by ChIP-qPCR in *wt* or *tho* (*mft1*Δ) mutant cells transformed with either an empty vector or the *GPD-hsRNH1* construct (+hsRNH1), and heat shocked at 37°C for 15min (% of immunoprecipitation; mean±SD, n=4 independent experiments). A strain expressing an untagged version of Nic96 (Nic96-myc: -) was used as a control. Statistical test: two-sided Mann-Whitney-Wilcoxon test; *, $p=2.86 \times 10^{-2}$. Note that Nic96 ChIP signals observed over control or intergenic loci vary slightly in conditions of altered R-loop levels, albeit in a non-significant manner, likely reflecting their proximity with one of the multiple NPC contact sites scored in our ChIP-seq analysis (more than 1000 peaks over the genome, Fig. 2e). Source data are provided as a Source Data file.

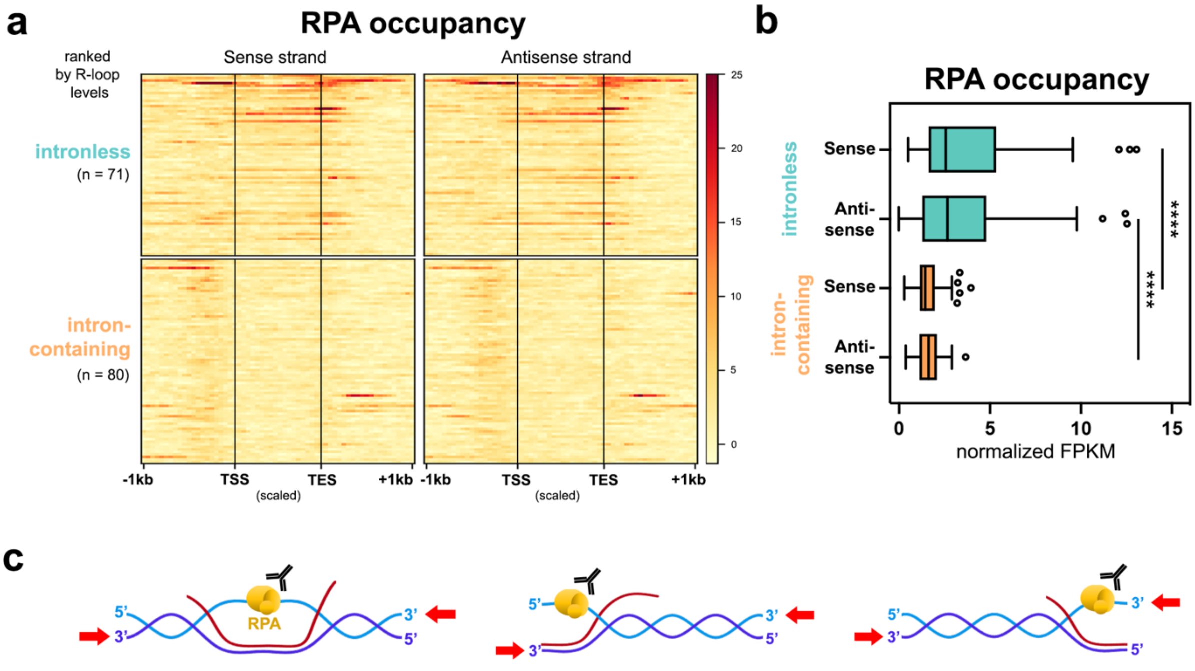

Supplementary Figure 3. Characterization of the R-loop gating pathway. a, Heatmap analysis of RPA occupancy at the sense or antisense strand of highly-transcribed intronless and intron-containing genes, aligned at their Transcription Start Site (TSS) and Transcription End Site (TES), in *wt* cells arrested in G1 (strand-specific RPA ChIP-seq dataset)[6]. Only the regions between the TSS and the TES are scaled. Genes are grouped based on their intron content and ranked according to their R-loop levels (from Fig. 1c). Note that the position of RPA tracks does not perfectly correlate with the localization of R-loops (Fig. 1c), raising the possibility that RPA could also associate to genomic loci in G1, independently of R-loop formation. **b,** Average RPA occupancy at the sense or antisense strand of highly-transcribed intronless and intron-containing genes (normalized to input DNA). Statistical test: two-sided Mann-Whitney-Wilcoxon test; ****, $p<10^{-4}$. **c,** Immunoprecipitation of R-loop-bound RPA is not expected to retrieve directional signals in strand-specific ChIP-seq. In the procedure used[6], immunoprecipitated DNA is denatured and 3'-specific adaptors are ligated (red arrows) prior to library amplification. Since R-loops are expected to be smaller (≈150bps)[7] than sonicated DNA fragments (200-500bp), this treatment will similarly tail both strands with the adaptors. Source data are provided as a Source Data file.

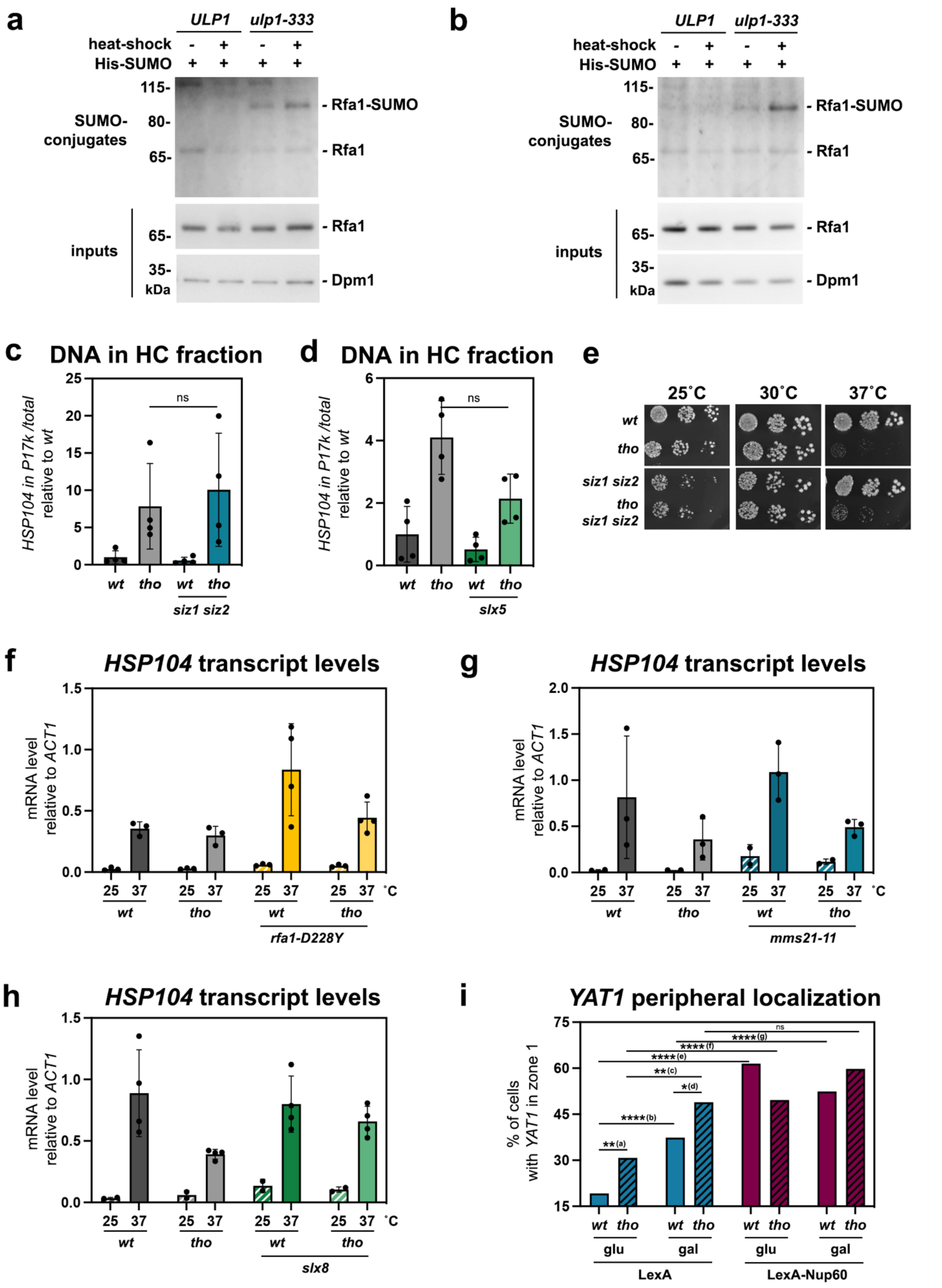

Supplementary Figure 4. Control experiments related to SUMOylation, fractionation, growth and tethering assays. See legend on next page.

Supplementary Figure 4. Control experiments related to SUMOylation, fractionation, growth and tethering assays. a-b, biological replicates of the SUMOylation assay featured in Fig. 4a. Rfa1 is detected by western-blot in input fractions (bottom panel) or purified SUMO-conjugates (top panel) obtained from the indicated strains. Wild type (*ULP1*) or *ulp1-333* cells carrying the His-SMT3 (His-SUMO) construct were grown at 25°C or heat shocked at 37°C for 15min (heat-shock). The positions of unmodified and mono-SUMOylated Rfa1 are indicated, as well as molecular weights (kDa, kilodaltons). Dpm1 serves as a loading control. **c-d**, qPCR-based quantification of the amount of DNA from the indicated loci in heavy chromatin (HC) fractions from the indicated strains heat-shocked at 37°C for 15min (% of *HSP104* in P17K relative to total [S17K+P17K]; mean±SD, n=4 independent experiments, relative to *wt*). Statistical test: two-sided Mann-Whitney-Wilcoxon test. **e,** Serial dilutions of the indicated strains were grown at the indicated temperatures on rich medium (YPD). **f-h,** qPCR-based quantification of *HSP104* mRNA levels from the indicated strains grown at 25°C or heat-shocked at 37°C for 15min (values normalised to *ACT1* RNA levels and relative to *wt*; mean±SD, n=2-3 independent experiments for 25°C and 3-4 for 37°C). **i,** Fraction of cells (%) showing intronless *YAT1* in zone 1, in *wt* or *tho* (*mft1*Δ) mutant cells carrying either LexA- or LexA-Nup60-expressing constructs, grown in glycerol-lactate medium and further treated with glucose or galactose for 5h. Statistical test: two-sided Fisher's exact test; P-values were calculated on the total number of counted cells (between 141 and 270 cells/condition); (a), p=8.22 x 10^{-3}; (b), p=7.24 x 10^{-6}; (c), p=1.34 x 10^{-3}; (d), p=2.43 x 10^{-2}; (e), p<10^{-8}; (f), p=2.18 x 10^{-4}; (g), p=5.95 x 10^{-4}. Source data are provided as a Source Data file.

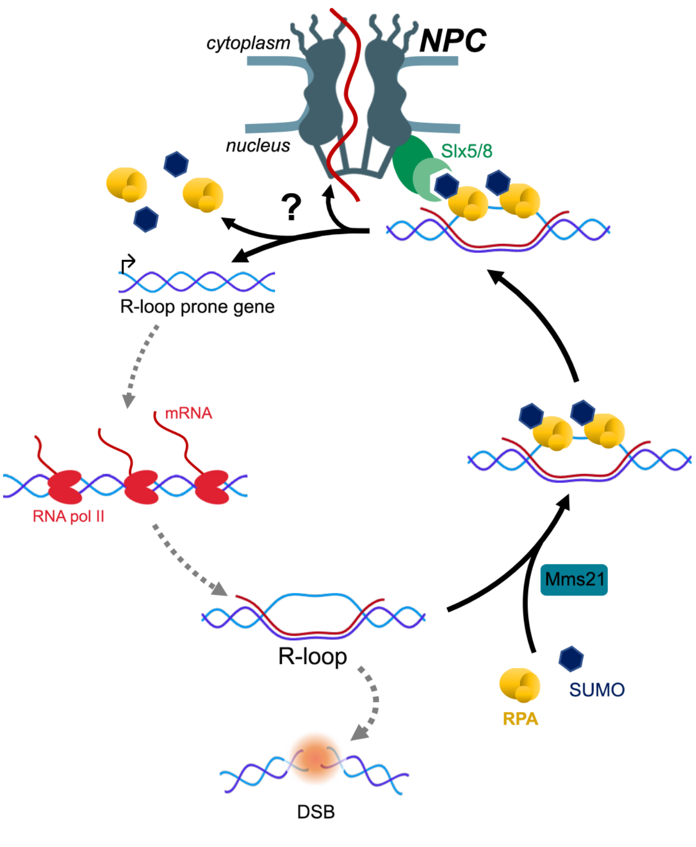

Supplementary Figure 5. Proposed model for the relocation of R-loop forming loci to nuclear pore complexes.

Supplementary Table 1. Intronless and intron-containing highly-expressed genes considered in this study.

Intronless (n=71)

Systematic Name	Standard Name	Systematic Name	Standard Name
YLR029C	RPL15A	YGR192C	TDH3
YLR110C	CCW12	YDR276C	PMP3
YJL158C	CIS3	YDR033W	MRH1
YLR044C	PDC1	YIL053W	GPP1
YKL060C	FBA1	YML028W	TSA1
YKL152C	GPM1	YHR193C	EGD2
YGL008C	PMA1	YNL031C	HHT2
YEL027W	VMA3	YDL055C	PSA1
YNL145W	MFA2	YJR009C	TDH2
YLR249W	YEF3	YGR234W	YHB1
YCR012W	PGK1	YEL009C	GCN4
YLR075W	RPL10	YLR441C	RPS1A
YDR382W	RPP2B	YBL003C	HTA2
YKL056C	TMA19	YGL147C	RPL9A
YOR063W	RPL3	YJL190C	RPS22A
YPL131W	RPL5	YBR010W	HHT1
YAL038W	CDC19	YDR134C	CCW22
YJR123W	RPS5	YDR225W	HTA1
YHL015W	RPS20	YDR418W	RPL12B
YOL086C	ADH1	YGL253W	HXK2
YOL040C	RPS15	YKL216W	URA1
YDR050C	TPI1	YLR300W	EXG1
YDR224C	HTB1	YLL045C	RPL8B
YGL123W	RPS2	YNL030W	HHF2
YDR461W	MFA1	YDR155C	CPR1
YOR369C	RPS12	YNL067W	RPL9B
YLR167W	RPS31	YLR264W	RPS28B
YDL081C	RPP1A	YDL192W	ARF1
YLR340W	RPP0	YEL054C	RPL12A
YOR167C	RPS28A	YGR037C	ACB1
YNL178W	RPS3	YPL037C	EGD1
YOL039W	RPP2A	YHR089C	GAR1
YOL109W	ZEO1	YDL014W	NOP1
YHR174W	ENO2	YEL034W	HYP2
YGR060W	ERG25	YBR106W	SND3
YLR325C	RPL38		

Intron-containing (n=80)

Systematic Name	Standard Name	Systematic Name	Standard Name
YGR034W	RPL26B	YHL001W	RPL14B
YLR061W	RPL22A	YNL302C	RPS19B
YAL003W	EFB1	YOR182C	RPS30B
YBL092W	RPL32	YIL133C	RPL16A
YGL103W	RPL28	YPR132W	RPS23B
YNL112W	DBP2	YPL143W	RPL33A
YGL030W	RPL30	YML026C	RPS18B
YDL130W	RPP1B	YML024W	RPS17A
YLR048W	RPS0B	YDL083C	RPS16B
YJL189W	RPL39	YLR388W	RPS29A
YPR043W	RPL43A	YIL148W	RPL40A
YBR048W	RPS11B	YJL177W	RPL17B
YOL127W	RPL25	YNL162W	RPL42A
YHR010W	RPL27A	YOL120C	RPL18A
YDL061C	RPS29B	YLR406C	RPL31B
YIL069C	RPS24B	YIL052C	RPL34B
YMR116C	ASC1	YOR096W	RPS7A
YHR021C	RPS27B	YDR447C	RPS17B
YGL031C	RPL24A	YLR185W	RPL37A
YDL075W	RPL31A	YLR333C	RPS25B
YML073C	RPL6A	YKR094C	RPL40B
YNL069C	RPL16B	YDR064W	RPS13
YIL018W	RPL2B	YDL082W	RPL13A
YJR145C	RPS4A	YDR471W	RPL27B
YDR500C	RPL37B	YDR025W	RPS11A
YKR057W	RPS21A	YLR448W	RPL6B
YBL087C	RPL23A	YBR189W	RPS9B
YGL189C	RPS26A	YOR312C	RPL20B
YBR191W	RPL21A	YKL156W	RPS27A
YPL079W	RPL21B	YNL301C	RPL18B
YGR118W	RPS23A	YCR031C	RPS14A
YGR148C	RPL24B	YMR143W	RPS16A
YJL136C	RPS21B	YBL027W	RPL19B
YOR234C	RPL33B	YHR141C	RPL42B
YHR203C	RPS4B	YER074W	RPS24A
YER117W	RPL23B	YGR214W	RPS0A
YKL180W	RPL17A	YNL096C	RPS7B
YOR293W	RPS10A	YMR142C	RPL13B
YDR450W	RPS18A	YLR344W	RPL26A
YOL121C	RPS19A	YMR230W	RPS10B

Supplementary Table 2. Strains used in this study.

CODE	NAME	GENOTYPE	USAGE (Fig.)	SOURCE
YBP539	*wt* (BY4742)	MATalpha *ura3 his3 leu2 lys2*	Fig. 1b-d, 2a-e, S2a-b, S2h-i, 3c, 4g, S4c-f, S4h, 5a	Euroscarf
YBP936	*wt* (BY4741)	MATa *ura3 his3 leu2 met15*	Fig. 3d	Euroscarf
YBP1525	*wt* (W303)	MATalpha *ade2 ura3 his3 trp1 leu2 can1*	Fig. 3b, 4a, 4d-f, S4a-b, S4g, 5a	Gift from C. Dargemont.
FSY3523	*wt* (*HSP104*)	(W303) *LacO@HSP104 3'::TRP1 ade2-1::GFP-LacI-ADE2 NUP49-GFP*	strain construction	Rougemaille et al.[8]
YKD2204	*wt* (*HSP104)*	(W303) *LacO@HSP104 3'::TRP1 ade2-1::GFP-LacI-ADE2 pRS316-NUP49-mCherry*	Fig. S2d, 3h, 4c	Derived from FSY3523 through crosses.
FSY5216	*wt* (*GAL10*)	(W303) *LacO@GAL10 3'::TRP1 his3::LacI-GFP::HIS3 LexA-BS@GAL1 3' NUP49-GFP + pBTM116-URAr-LexA*	strain construction	Texari et al.[9]
YKD1683	*wt* (*GAL10*)	(W303) *LacO@GAL10 3'::TRP1 his3::LacI-GFP::HIS3 LexA-BS@GAL1 3' NUP49::NUP49-mCherry-HphMX*	strain construction	Derived from FSY5216 through crosses.
YPB2102	*wt* (*YAT1)*	(W303) *LacO@GAL10 3'::TRP1 his3::LacI-GFP::HIS3 LexA-BS@GAL1 3' NUP49::NUP49-mCherry-HphMX leu2∆3'- GAL1$_{prom}$-YAT1-leu2∆5'::KanMX@GAL1 3'*	Fig. 1h-i, S1f-h, S2g, 5c, S4i	Obtained by homologous recombination into YKD1683 using a cassette encompassing *leu2* repeats and the *YAT1* transgene, retrieved from pBP2057 by PacI-PmeI restriction and further integrated at *GAL1 3'*, upstream LexA-BS.
YPB2103	*wt* (*intron-YAT1)*	(W303) *LacO@GAL10 3'::TRP1 his3::LacI-GFP::HIS3 LexA-BS@GAL1 3' NUP49::NUP49-mCherry-HphMX leu2∆3'- GAL1$_{prom}$ -intron-YAT1-leu2∆5'::KanMX@GAL1 3'*	Fig. 1i, S1f-g	Obtained by homologous recombination into YKD1683 using a cassette encompassing *leu2* repeats and the intron-containing *YAT1* transgene, retrieved from pBP2060 by PacI-PmeI restriction and further integrated at *GAL1 3'*, upstream LexA-BS.
YBP1501	*tho* (BY4742)	(BY4742) *mft1::KanMX*	Fig. 2c-e, S2b, S2h-i, 3c, 4g, S4c-f, S4h, 5a,	Euroscarf
YBP2109	*tho* (BY4741)	(BY4741) *mft1::KanMX*	Fig. 3d	Euroscarf
YPB2006	*tho* (W303)	(W303) *mft1::KanMX*	Fig. 4d-f, S4g, 5a	The complete *MFT1* CDS was deleted by homologous recombination using a KanMX cassette amplified from pFA6a-KanMX6.
YKD2373	*tho* (*HSP104)*	(YKD2204) *mft1::KanMX*	Fig. S2d, 3h, 4c	The complete *MFT1* CDS was deleted by homologous recombination using a KanMX cassette amplified from pFA6a-KanMX6.
YKD2564	*nup133* (*HSP104)*	(YKD2204) *nup133::URA3*	Fig. S2e-f	The complete *NUP133* CDS was deleted by homologous recombination using a *nup133::URA3* cassette[10].

YBP2104	*tho* (*GAL10*)	(YKD1683) *mft1::*NatMX	strain construction	The complete *MFT1* CDS was deleted by homologous recombination using a NatMX cassette amplified from pFA6a-NatMX6.
YBP2105	*tho* (*YAT1)*	(W303) *LacO@GAL10 3'::TRP1 his3::LacI-GFP::HIS3 LexA-BS@GAL1 3' NUP49::NUP49-mCherry-HphMX leu2Δ3'-GAL1$_{prom}$-YAT1-leu2Δ5'::KanMX@GAL1 3' mft1::NatMX*	Fig. 1i, S1f-g, S2g, 5c, S4i	Obtained by homologous recombination into YBP2104 using a cassette encompassing *leu2* repeats and the *YAT1* transgene, retrieved from pBP2057 by PacI-PmeI restriction and further integrated at *GAL1 3'*, upstream LexA-BS.
YBP2106	*tho* (*intron-YAT1)*	(W303) *LacO@GAL10 3'::TRP1 his3::LacI-GFP::HIS3 LexA-BS@GAL1 3' NUP49::NUP49-mCherry-HphMX leu2Δ3'- GAL1$_{prom}$ -intron-YAT1-leu2Δ5'::KanMX@GAL1 3' mft1::NatMX*	Fig. 1i, S1f-g,	Obtained by homologous recombination into YBP2104 using a cassette encompassing *leu2* repeats and the intron-containing *YAT1* transgene, retrieved from pBP2060 by PacI-PmeI restriction and further integrated at *GAL1 3'*, upstream LexA-BS.
YBP2307	*NIC96-myc*	(BY4742) *NIC96-13Myc::KanMX*	Fig. 1b-d, 2d-e, S2i, 3e-f	C-terminal tagging of Nic96 was achieved through the integration of a 13Myc-KanMX cassette, amplified from pFA6a-13Myc-KanMX6, at the endogenous *NIC96* locus. Nic96-myc expression is driven by *NIC96* natural promoter.
YBP2308	*tho NIC96-myc*	(BY4742) *NIC96-13Myc::KanMX mft1::NatMX*	Fig. 2d-e; S2i, 3f	The complete *MFT1* CDS was deleted by homologous recombination using a NatMX cassette amplified from pFA6a-NatMX6.
YBP2409	*sac3Δ*	(W303) *sac3::KanMX*	Fig. 3b	The complete *SAC3* CDS was deleted by homologous recombination using a KanMX cassette amplified from pFA6a-KanMX6.
YBP2326	*fcy1Δ*	(BY4742) *fcy1::KanMX*	strain construction	Euroscarf
YBP2327	*mlh3Δ*	(BY4742) *mlh3::KanMX*	strain construction	Euroscarf
YBP2017	*rad2Δ*	(BY4742) *rad2::KanMX*	strain construction	Euroscarf
YBP2161	*tho* (BY4741)	(BY4741) *mft1::NatMX*	strain construction	The complete *MFT1* CDS was deleted by homologous recombination using a NatMX cassette amplified from pFA6a-NatMX6.
YBP2332	*tho fcy1Δ*	(BY4742) *fcy1::KanMX mft1::NatMX*	Fig. 3c	Obtained by cross (YBP2326xYBP2161).
YBP2331	*tho mlh3Δ*	(BY4742) *mlh3::KanMX mft1::NatMX*	Fig. 3c	Obtained by cross (YBP2327xYBP2161).
YBP2018	*tho rad2Δ*	(BY4742) *rad2::KanMX mft1::KanMX*	Fig. 3c	Obtained by cross (YBP2017xYBP2161).
YBP2315	*NIC96-myc rfa1-D228Y*	(YBP2307) *rfa1-D228Y::NatMX*	Fig. 3e-f	Obtained by homologous recombination into YBP2307 using a cassette encompassing the *RFA1* CDS carrying the D228Y mutation and the *NatMX* marker, retrieved from pBP2153 by NotI restriction and further integrated at the *RFA1 locus.*
YBP2319	*tho NIC96-myc rfa1-D228Y*	(YBP2308) *rfa1-D228Y::NatMX*	Fig. 3e-f	Obtained by cross (YBP2109xYBP2315).
YBP1478	*siz1Δ siz2Δ*	(BY4742) *siz1::KanMX siz2::KanMX*	Fig. S4c, S4e	Bretes et al.[11]
YBP2238	*tho siz1Δ siz2Δ*	(BY4742) *siz1::KanMX siz2::KanMX mft1::NatMX*	Fig. S4c, S4e	Obtained by cross (YBP1478xYBP2161).

YBP2277	*slx5Δ*	(BY4742) *slx5*::*NatMX*	Fig. S4d	The complete *SLX5* CDS was deleted by homologous recombination using a NatMX cassette amplified from pFA6a-NatMX6.
YBP2237	*tho slx5Δ*	(BY4742) *slx5*::*NatMX mft1::KanMX*	Fig. S4d	Obtained by cross (YBP2277xYBP2109).
YBP1167	*slx8Δ*	(BY4742) *slx8::HphMX*	Fig. 4g, 5a, S4h	The complete *SLX8* CDS was deleted by homologous recombination using a HphMX cassette amplified from pFA6a-HphMX6.
YBP2166	*tho slx8Δ*	(BY4742) *slx8::HphMX mft1::NatMX*	Fig. 4g, 5a, S4h	Obtained by cross (YBP1167xYBP2161).
YBP2324	*rfa1-D228Y*	(BY4742) *rfa1-D228Y*	Fig. 5a, S4f	Obtained by cross (YBP2109xYBP2315).
YBP2325	*tho rfa1-D228Y*	(BY4742) *rfa1-D228Y mft1::KanMX*	Fig. 5a, S4f	Obtained by cross (YBP2109xYBP2315).
FSY3992	*ulp1-333*	(W303) *ULP1::HIS3 YCpLac22-ulp1-333-TRP1*	Fig. 4a, S4a-b	Infantino et al.[12]
Z417-17	*rfa1-4KR*	(W303) *rfa1-* K170R, K180R, K411R, K427R *mft1::KanMX*	Fig. 4f	Dhingra et al.[13]
YBP2333	*ulp1-333 rfa1-4KR*	(W303) *rfa1-* K170R, K180R, K411R, K427R; *ULP1::KanMX; YCpLac11-LEU2-ulp1-333*	Fig. 4a	The complete *ULP1* CDS was deleted by homologous recombination using a KanMX cassette (amplified from pFA6a-KanMX6) into Z417-17 cells carrying the *YCpLac11-LEU2-ulp1-333* plasmid.
YBP2280	*tho rfa1-4KR*	(W303) *rfa1-* K170R, K180R, K411R, K427R *mft1::KanMX*	Fig. 4f	The complete *MFT1* CDS was deleted by homologous recombination using a KanMX cassette amplified from pFA6a-KanMX6.
T79-9	*mms21-11*	(W303) *mms21-11*-LEU2	Fig. 4d, 5a, S4g	Gift from X. Zhao
YBP2278	*tho mms21-11*	(W303) *mms21-11*-LEU2 *mft1::KanMX*	Fig. 4d, 5a, S4g	Obtained by cross (T79-9x[*mft1Δ* mat a W303]).
YBP2282	*smt3-3KR*	(W303) *smt3- K11R, K15R, K19R::TRP1*	Fig. 4e	Lescasse et al.[14]
YBP2290	*tho smt3-3KR*	(W303) *smt3- K11R, K15R, K19R::TRP1 mft1::KanMX*	Fig. 4e	Obtained by cross (YBP2282x[*mft1Δ* mat a W303]).
MNY1125	*rfa1-D228Y*	(W303) *rfa1-D228Y*	strain construction	Luciano et al.[15]
YKD2206	*rfa1-D228Y (HSP104)*	(YKD2204) *rfa1-D228Y*	Fig. 3h	Derived from YKD2204 and MNY1125 by successive crosses.
YKD2338	*tho rfa1-D228Y (HSP104)*	(YKD2204) *rfa1-D228Y mft1::KanMX*	Fig. 3h	The complete *MFT1* CDS was deleted by homologous recombination using a KanMX cassette amplified from pFA6a-KanMX6.
YKD2426	*rfa1-4KR (HSP104)*	(YKD2204) *rfa1-* K170R, K180R, K411R, K427R	Fig. 4c	Derived from YKD2204 and Z417-17 by successive crosses.
YKD2463	*tho rfa1-4KR (HSP104)*	(YKD2204) *rfa1-* K170R, K180R, K411R, K427R *mft1::KanMX*	Fig. 4c	The complete *MFT1* CDS was deleted by homologous recombination using a KanMX cassette amplified from pFA6a-KanMX6.
YKD2372	*mms21-11 (HSP104)*	(YKD2204) *mms21-11::LEU2*	Fig. 4c	Obtained from YKD2204 and T79-9 by successive crosses.
YKD2375	*tho mms21-11 (HSP104)*	(YKD2204) *mms21-11::LEU2 mft1::KanMX*	Fig. 4c	The complete *MFT1* CDS was deleted by homologous recombination using a KanMX cassette amplified from pFA6a-KanMX6.

Supplementary Table 3. Plasmids used in this study.

CODE	NAME	RELEVANT FEATURES	SOURCE	USAGE (Fig.)
pBP414	*pFA6a-KanMX6*	AmpR; TEF_{prom}-KanMX6-TEF_{term}	Longtine et al.[16]	Strain construction
pBP679	*pFA6a-NatMX6*	AmpR; TEF_{prom}-NatMX6-TEF_{term}	Hentges et al.[17]	Strain construction
pBP672	*pFA6a-HphMX6*	AmpR; TEF_{prom}-HphMX6-TEF_{term}	Hentges et al.[17]	Strain construction
pBP780	*pFA6a-13Myc-KanMX6*	AmpR; 13Myc-TEF_{prom}-KanMX6-TEF_{term}	Longtine et al.[16]	Strain construction
pBP1172	*YCpLac11-LEU2-ulp1-333*	AmpR/*LEU2/CEN; $ULP1_{prom}$-ulp1-333*	Bretes et al.[11]	Strain construction
pBP2057	*pFA6a-YAT1 **	AmpR; *GAL1 3'- leu2Δ3'-pGAL1-YAT1-leu2Δ5'::KanMX-GAL1 3'*	This Study	Strain construction
pBP2060	*pFA6a-intron-YAT1 **	AmpR; *GAL1 3'- leu2Δ3'-pGAL1-RPL51A*intron-YAT1-leu2Δ5'::KanMX- GAL1 3'*	This Study	Strain construction
pBP2153	*pRS315-rfa1-D228Y-NatMX*	AmpR/*LEU2/CEN*; $rfa1_{D228Y}$-NatMX6-RFA1 3'	This Study	Strain construction
pBP2182	*pRS315-TET-off scRNH1-Flag*	AmpR/*LEU2/CEN*; CMV_{prom}-TetR-VP16, $tetO_7$-scRNH1-3xFlag	This study	Fig. 2a; S2b
pBP1932	*pRS423-GPD-hsRNH1*	AmpR/*HIS3/2μ*; GPD_{prom}-myc-hsRNH1-$CYC1_{term}$	Bonnet et al.[3]	Fig. 2c, S2h-i
pBP1026	*pRS316-NUP49-mCherry*	AmpR/*URA3/CEN*; $NUP49_{prom}$-NUP49-mCherry	Chadrin et al.[18]	Fig. 3h, 4c, S2d-f
pBP2155	*pRS316-His_6-SMT3*	AmpR/*URA3/CEN*; $SMT3_{prom}$-His6-SMT3-$SMT3_{term}$	This study	Fig. 4a, S4a-b
pBP1882	*pBTM116-URA-LexA*	AmpR/*URA3/2μ*; $ADH1_{prom}$-LexA	Texari et al. [9]	Fig. 5c, S4i
pBP1883	*pBTM116-URA-LexA-Nup60*	AmpR/*URA3/2μ*; $ADH1_{prom}$-LexA-NUP60	Texari et al. [9]	Fig. 5c, S4i

* *YAT1* reporter cassettes encompass: (i) a 100bp DNA fragment homologous to *GAL1 3'* (133-232bp downstream *GAL1* stop codon); (ii) the *leu2Δ3'-$GAL1_{prom}$-YAT1-leu2Δ5'* or *leu2Δ3'-$GAL1_{prom}$-RPL51A*intron-YAT1-leu2Δ5'* reporter constructs, as previously defined[3]; (iii) the KanMX selection marker; (iv) a 100bp DNA fragment homologous to *GAL1 3'* (234-333bp downstream *GAL1* stop codon). Homologous recombination allows the integration of the cassette at *GAL1* 3' (233bp downstream the stop codon), upstream LexA binding sites (inserted 334bp downstream the stop codon in the FSY5216 parental strain[9]).

Supplementary Table 4. Sequences of oligonucleotides used in this study.

***HSP104* F**	GTTCTACCAAATCACGAAGC
***HSP104* R**	TCTAGGTCATCATCAATTTCC
***YEF3* F**	GATTGCCGGTGGTAAGAAGA
***YEF3* R**	CGTAAGCATCACCCAATTCC
**Intergenic* F**	GAAACCACGAAAAGTTCACCA
**Intergenic* R**	AGCTTCTGCAAACCTCATTTG
***YAT1* F**	TCTGTGGTGGTGTCCTCAAG
***YAT1* R**	CTTGCTGCCGTTTGAAGATG
***ACT1* F**	ACGTTACCCAATTGAACACG
***ACT1* R**	AGAACAGGGTGTTCTTCTGG
***GSY2* F**	ATGACCCCTGGTGATTTGGG
***GSY2* R**	TCAGCATATGGGCCATCGTC
***PAU17* F**	CCCCGCTGACCAAGTCACTA
***PAU17* R**	AGCAGTGTAGATACCGTCTGC
***SSA4* F**	TGGAATCCATTGCTTACTCTTTGA
***SSA4* R**	AGCCTTCTTAGTGACAGCGT

***** chrIV:43199..53262

Supplementary References

1. Pelechano, V., Chávez, S. & Pérez-Ortín, J. E. A complete set of nascent transcription rates for yeast genes. *PloS One* **5**, e15442 (2010).
2. Wahba, L., Costantino, L., Tan, F. J., Zimmer, A. & Koshland, D. S1-DRIP-seq identifies high expression and polyA tracts as major contributors to R-loop formation. *Genes Dev.* **30**, 1327–1338 (2016).
3. Bonnet, A. *et al.* Introns Protect Eukaryotic Genomes from Transcription-Associated Genetic Instability. *Mol. Cell* **67**, 608-621.e6 (2017).
4. Aiello, U. *et al.* Sen1 is a key regulator of transcription-driven conflicts. *Mol. Cell* S1097-2765(22)00604–9 (2022) doi:10.1016/j.molcel.2022.06.021.
5. Mouaikel, J. *et al.* High-frequency promoter firing links THO complex function to heavy chromatin formation. *Cell Rep.* **5**, 1082–1094 (2013).
6. Reusswig, K.-U. *et al.* Unscheduled DNA replication in G1 causes genome instability and damage signatures indicative of replication collisions. *Nat. Commun.* **13**, 7014 (2022).
7. García-Pichardo, D. *et al.* Histone Mutants Separate R Loop Formation from Genome Instability Induction. *Mol. Cell* **66**, 597-609.e5 (2017).
8. Rougemaille, M. *et al.* THO/Sub2p Functions to Coordinate 3′-End Processing with Gene-Nuclear Pore Association. *Cell* **135**, 308–321 (2008).
9. Texari, L. *et al.* The Nuclear Pore Regulates GAL1 Gene Transcription by Controlling the Localization of the SUMO Protease Ulp1. *Mol. Cell* **51**, 807–818 (2013).
10. Belgareh, N. & Doye, V. Dynamics of nuclear pore distribution in nucleoporin mutant yeast cells. *J. Cell Biol.* **136**, 747–759 (1997).
11. Bretes, H. *et al.* Sumoylation of the THO complex regulates the biogenesis of a subset of mRNPs. *Nucleic Acids Res.* **42**, 5043–5058 (2014).
12. Infantino, V. *et al.* The mRNA export adaptor Yra1 contributes to DNA double-strand break repair through its C-box domain. *PloS One* **14**, e0206336 (2019).
13. Dhingra, N., Wei, L. & Zhao, X. Replication protein A (RPA) sumoylation positively influences the DNA damage checkpoint response in yeast. *J. Biol. Chem.* **294**, 2690–2699 (2019).

14. Lescasse, R., Pobiega, S., Callebaut, I. & Marcand, S. End-joining inhibition at telomeres requires the translocase and polySUMO-dependent ubiquitin ligase Uls1. *EMBO J.* **32**, 805–815 (2013).
15. Luciano, P. *et al.* RPA facilitates telomerase activity at chromosome ends in budding and fission yeasts. *EMBO J.* **31**, 2034–2046 (2012).
16. Longtine, M. S. *et al.* Additional modules for versatile and economical PCR-based gene deletion and modification in Saccharomyces cerevisiae. *Yeast Chichester Engl.* **14**, 953–961 (1998).
17. Hentges, P., Van Driessche, B., Tafforeau, L., Vandenhaute, J. & Carr, A. M. Three novel antibiotic marker cassettes for gene disruption and marker switching in Schizosaccharomyces pombe. *Yeast Chichester Engl.* **22**, 1013–1019 (2005).
18. Chadrin, A. *et al.* Pom33, a novel transmembrane nucleoporin required for proper nuclear pore complex distribution. *J. Cell Biol.* **189**, 795–811 (2010).
